# Computer International Standards for Neurological Classification of Spinal Cord Injury (ISNCSCI) algorithms: a review

**DOI:** 10.1038/s41393-022-00854-2

**Published:** 2022-09-16

**Authors:** Kristen Walden, Christian Schuld, Vanessa K. Noonan, Rüdiger Rupp

**Affiliations:** 1 Praxis Spinal Cord Institute, Vancouver, BC Canada; 2 Spinal Cord Injury Center, Heidelberg University Hospital, Heidelberg, Germany; 3 International Collaboration on Repair Discoveries, Vancouver, BC Canada

**Keywords:** Spinal cord diseases, Outcomes research, Physical examination, Spinal cord

## Abstract

**Study design:**

Literature review and survey.

**Objectives:**

To provide an overview of existing computerized International Standards for Neurological Classification of Spinal Cord Injury (ISNCSCI) algorithms and to evaluate the use of the current algorithms in research and clinical care.

**Setting:**

Not applicable.

**Methods:**

Literature review according to three organizing concepts for evaluation of Health Information Products (reach, usefulness, and use) was conducted.

**Results:**

While the use of computerized ISNCSCI algorithms has been around for many years, many were developed and used internally for specific projects or not maintained. Today the International SCI community has free access to algorithms from the European Multicenter Study about Spinal Cord Injury (EMSCI) and the Praxis Spinal Cord Institute. Both algorithms have been validated in large datasets and are used in different SCI registries for quality control and education purposes. The use of the Praxis Institute algorithm by clinicians was highlighted through the Praxis User Survey (*n* = 76) which included participants from 27 countries. The survey found that over half of the participants using the algorithm (*N* = 69) did so on a regular basis (51%), with 54% having incorporated it into their regular workflow.

**Conclusions:**

Validated computerized ISNCSCI classification tools have evolved substantially and support education, clinical documentation, communication between clinicians and their patients, and ISNCSCI data quality around the world. They are not intended to replace well-trained clinicians, but allow for reclassification of ISNCSCI datasets with updated versions of the ISCNSCI, and support rapid classification of large datasets.

## Introduction

The International Standards for Neurological Classification of Spinal Cord Injury (ISNCSCI) exam is the gold standard assessment used to determine the level and severity of neurological injury after spinal cord injury (SCI). Originally developed in 1982, the ISNCSCI is defined by the International Standards Committee of the American Spinal Cord Injury Association (ASIA), and continues to undergo regular revisions. Now in its eighth edition [1], the ISNCSCI represents an important tool for both clinical care and research [2, 3].

There are two components to obtaining an accurate and reliable ISNCSCI exam: the first is performing the bedside examination to obtain motor, sensory and rectal exam scores. The second is using those scores to classify the SCI using the ISNCSCI classification rules [4]. These classification rules are used to determine total sensory and upper and lower extremity motor scores, sensory and motor levels as well as a single neurological level of injury, the ASIA Impairment Scale (AIS) together with a broad categorization of the severity of injury (complete/incomplete) and –if applicable– the zones of partial preservation (ZPPs). It has been shown that training can improve the performance of the bedside exam, as well as classification [5, 6].

Despite training, error-free classification remains an issue and has been a primary driver for the development of computerized algorithms that can perform the classification using a standardized set of ISNCSCI rules [7–12]. The opportunity to implement ISNCSCI classification algorithms as a computer program was recognized and many computerized ISNCSCI algorithms have been developed over the last two decades. The purpose of this paper is to provide an overview of existing computerized ISNCSCI algorithms and evaluate the current algorithms available for use in research and clinical care and provide recommendations for future directions.

## Methods

### Literature overview of computerized ISNCSCI algorithms

**Fig. 1:**
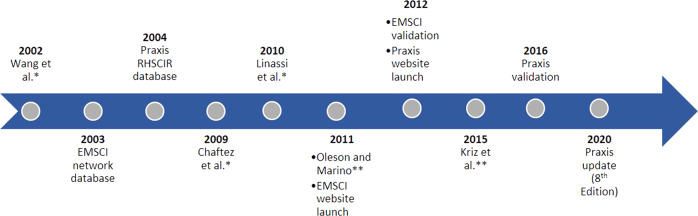
Timeline of computerized ISNCSCI algorithm development. EMSCI European Multicenter Study about Spinal Cord Injury; RHSCIR Rick Hansen Spinal Cord Injury Registry. *No known follow-up; **Developed for internal project quality control purposes only.

The first computerized ISNCSCI algorithm was published by Wang et al. [10], presented at the joint meeting of the ASIA and the International Spinal Cord Society (ISCoS) in 2002, and there have been 7 in total presented and/or published as of 2021 (see Fig. 1). Most of these computerized ISNCSCI algorithms were developed for specific research projects and many [7, 10–13] have been presented or published without any known or published follow-up. Reasons for the original development of these algorithms include: SCI clinical research data quality control [8, 9, 11, 12]; improving and standardizing clinical ISNCSCI use [7, 13]; reducing the time required for classification and documentation of the ISNCSCI exam both for individual exams [7] and large datasets [8]; and supporting education [7–9].

There are two ISNCSCI algorithms that are publicly available and updated to reflect the 2011 or 2019 versions of the Standards. The European Multicenter Study about Spinal Cord Injury (EMSCI) network has included an ISNCSCI classification algorithm since 2003 [14].The algorithm has been validated using a dataset (*N* = 5542) from the EMSCI network (Table 1) [8]. The second is the Praxis (formerly known as the Rick Hansen Institute) ISNCSCI algorithm developed as part of the Rick Hansen SCI Registry (RHSCIR) database [15] in 2004. It was redeveloped in collaboration with ISCoS and a group of international experts in 2012 and was made publicly available at the ISCoS and the Academy of Spinal Cord Injury Professionals (ASCIP) meetings in 2012 [16, 17]. The 2012 version was validated using input from the group of international experts as well as the SCI community along with a dataset of 2106 ISNCSCI cases from the RHSCIR [9].

**Table 1:** Overview of EMSCI and Praxis ISNCSCI computerized algorithms.

	EMSCI ISNCSCI calculator	Praxis ISNCSCI algorithm
Web application public release date	2012	2012 (Beta test version)/2014 Final 1.0 version/2020 Version 2.0 (Eighth edition updates)
Development team	Christian Schuld Ruediger Rupp	Praxis IT Team Praxis Clinical staff International Expert Advisory Group
ISNCSCI version	Seventh edition/2011 version of ISNCSCI	Eighth edition/2019 version of ISNCSCI
Programming language	C, C++, JavaScript	V1.0: C#; V2.0: TypeScript
Validation	Validated using 2011 ISNCSCI Standards	• Previous versions validated using 2011 ISNCSCI Standards • Version 2.0 validated using 2019 ISNCSCI Standards
Clinical database used for validation	European Multicenter Study about SCI	Rick Hansen Spinal Cord Injury Registry
Expressed limitations	• Not recommended for use in lesions with multiple spinal injury levels • Not intended to replace clinical judgement or the performance of manual classification/classification review by an experienced examiner	
Data entry required	Motor, sensory and rectal bedside examination scores including lowest of non-key muscle segmental levels with motor function if applicable	
Classification provided	Motor and sensory levels, neurological level of injury, total motor and sensory scores, ASIA Impairment Scale (AIS), severity of injury (complete/incomplete), zones of partial preservation (ZPPs) if applicable	
		For classification variables that are not determinable (ND), the range of possible values is also displayed
Data output formats	CSV file PDF ISNCSCI worksheet Webpage with color coded myotome and dermatome visualizations (Fig. 2C)	ISNCSCI worksheet – PDF, image (PNG), or Excel (.xlsx) file
Feedback mechanism		‘Feedback’ button allows users to provide general feedback and obtain further explanation for classification where their classification did not align with that determined by the algorithm
Language(s)	English, Chinese, Czech	English, Chinese^a^
Availability	Public web application	Public web application Source code freely available in an open source format (Apache License, Version 2.0)

^a^V1.0 only.

**Fig. 2:**
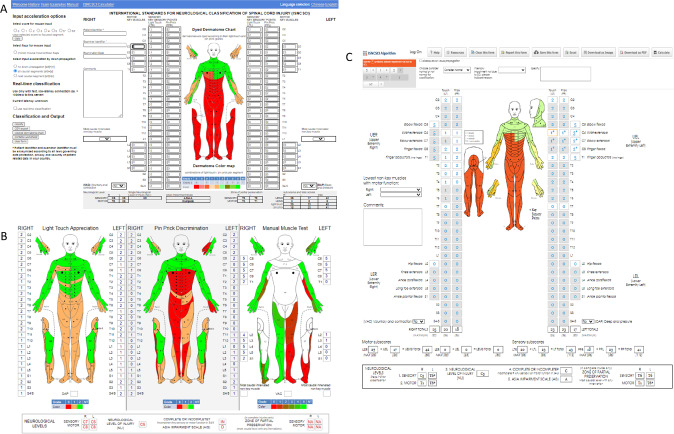
Web application interface of the two identified ISNCSCI algorithms depicting cases with differing results. A case with normal sensory functions rostral to and at C5, normal motor function C5-T1 and normal sensory function in T2-3 on the right side of the body, and with a brachial plexus injury on the left side of the body resulting in abnormal motor and sensory scores from C6-C8. **A** depicts the EMSCI ISNCSCI calculator classification results: Right sensory level C5, right motor level T3; Left sensory level C5, left motor level C6. **B** Depicts visualization from the EMSCI ISNCSCI calculator showing a Brown-Séquard syndrome example (this clinical syndrome is characterized by an asymmetrical loss of strength and a more severe impairment of pin prick sensation on the side of the body with less motor impairments. This neurological condition can be quickly identified as green right leg in the muscle test visualization and dark red right leg in the pin prick sensation visualization). **C** Depicts the Praxis ISNCSCI algorithm with results based on the new use of the asterisk (*) in the updated ISNCSCI eighth edition classification rules: Right sensory level C5, right motor level T1; Left sensory level T6*, left motor level T6*.

Currently the EMSCI algorithm classifies using the 2011 version of the ISNCSCI and Praxis algorithm classifies according to the 2019 version. Made publicly available via web application interfaces (Fig. 2A, C) in 2011 (EMSCI) and 2012 (Praxis), these computer algorithms share many of the same features [18, 19]. Developed to support education and quality control for the EMSCI and RHSCIR databases, both require entry of the clinically determined sensory and motor scores and the anorectal exam results, using automated logic to determine the resulting classification variables. Both support the classification of cases with not testable (NT) scores, where the manual classification might be challenging. Table 1 provides an overview of these computer algorithms, including both similarities and differences.

Since initial development, these two computer algorithms have both undergone improvements and continue to make updates to improve usability and outputs. Originally meant for demonstration purposes, the EMSCI ISNCSCI calculator has incorporated data export interfaces [20], multiple application programming interfaces [20], and extended visualizations [21] (Fig. 2B). The initial Praxis ISNCSCI algorithm incorporated the use of an exclamation mark to enable classification with cases where there was a non-SCI condition causing motor or sensory weakness above the neurological level of injury, which has now been updated to reflect asterisk use as outlined in the updated eighth edition [22]. Additionally, it provides the algorithm source code for both V1.0 (seventh ISNCSCI edition) and V2.0 (eighth ISNCSCI edition) in an open source format to facilitate integration into other applications. While in the majority of cases the results of both algorithms match, there are unique cases where the algorithms get different classification results. These rare cases have in common that the sensory level is within a segment with testable key muscles (C5-T1 or L2-S1), the key muscle functions of the respective extremity are all normal and a region with normal sensory functions is found caudal to the clinically testable myotomes of the motor intact extremity (Fig. 2A, C) [23]. This type of cases with differing classification results of the two algorithms help to identify parts of ISNCSCI where further clarifications are needed. These were therefore shared with ASIA’s International Standards Committee for discussion and to inform future revisions or updates of the standards.

#### Evaluation of the EMSCI and Praxis ISNCSCI algorithms in research and care

To better understand the reach and impact of these publicly available algorithms in both research and care, a review was performed using Sullivan, Strachan and Timmons (2007) three organizing concepts for evaluation of Health Information Products: reach, usefulness, and use [24].

##### Reach

(defined as the breadth and saturation of product dissemination), incorporating: distribution, both directly or indirectly (e.g. web application use), and referrals by other projects [24] was determined in April 2021 by the number and type of reported users (e.g. from citations, requests for source code use); number of languages the web application is available in; and rates of citation for the validation papers of the two algorithms [8, 9] in the peer-reviewed academic literature obtained from Google Scholar on April 19, 2021. For the Praxis algorithm, distribution was also measured through web application analytics between August 1, 2012-February 28, 2021.

##### Usefulness

(defined as the quality of information products and services that is appropriate, applicable and practical) can be largely determined by user satisfaction and perceived quality of a product [24]. Perceived quality was evaluated by reviewing algorithm validation and existing literature citing either algorithm validation paper for quality related reports. User satisfaction was evaluated using 14 questions from a Praxis ISNCSCI algorithm User Survey on use and usefulness which was open between 2016 and 2017. Questions were asked about the users’ profession, method and frequency of use, what the algorithm was being used for, challenges during use, and specific impacts of use.

##### Use

(defined as what is done with knowledge gained from the information product or service) incorporates both the amount of use and context of use [24]. Use was measured by reviewing reported uses in both literature citing these algorithms (April 2021) and ongoing clinical studies, Praxis User Survey results for questions about frequency and context of use, and outlining algorithm use to date by the ASIA International Standards Committee.

## Results

### Reach

EMSCI and Praxis computerized ISNCSCI algorithms have been integrated into multiple platforms for users. Both are available through public web applications and accessed internationally. Statistics are not available for the EMSCI ISNCSCI Calculator web application due to European General Data Protection Regulations. It is available in three languages (see Table 1) [25]. The EMSCI algorithm validation publication (2012) has been cited 38 times (Google scholar accessed 19 April 2021), including twenty-one related to clinical research, six to the ISNCSCI itself (e.g. challenging cases, training, etc.), four to clinical practice or SCI registries, two to ISNCSCI algorithms, one that referenced the algorithm to show the uncertainty of early ISNCSCI exams, and four either not available in English or that did not describe the ISNCSCI algorithm use.

Between August 1st, 2012 and February 28, 2021, the Praxis ISNCSCI algorithm web application was accessed 207,994 times by 114,323 users in 175 countries (web application Google statistics accessed April 20, 2021). It is available in two languages (see Table 1). The freely available open source code has been downloaded 2174 times. The Praxis algorithm validation publication has been cited 32 times (Google scholar accessed 19 April 2021), including twenty relating to clinical research, nine to clinical practice or SCI registries/harmonized datasets, one to ISNCSCI algorithms, and two that did not describe the ISNCSCI algorithm use.

### Usefulness

In terms of the perceived quality, both EMSCI and Praxis algorithms have been validated for determining ISNCSCI classification in a variety of real cases including those with not testable values (EMSCI *N* = 5542 exams from EMSCI database; Praxis *N* = 2106 exams from RHSCIR) [8, 9]. The EMSCI algorithm has also been found to reduce the time required for classification and documentation of the ISNCSCI exam, both in individual exams as well as large datasets [8]. Literature citing the algorithms reports they improve accuracy by reducing clinician determined classification errors [14, 26, 27]. The Praxis algorithm is referenced as a valuable tool to be included in the standardization of data for clinical use and research in SCI, and the use of an ISNCSCI algorithm is also recommended to characterize natural recovery after SCI [28, 29]. In addition, Dvorak et al. propose using these algorithms to help improve the accuracy of neurological assessments required for informing care and research [30].

**Fig. 3:**
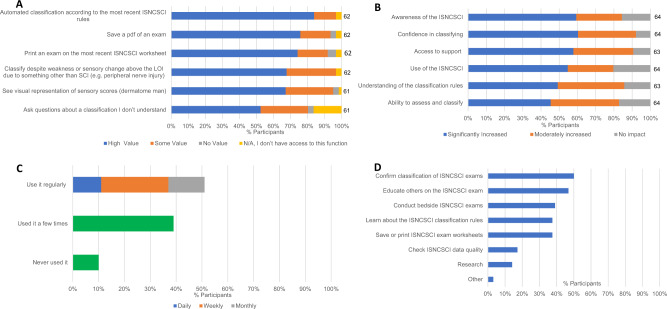
Use and usefulness of the Praxis ISNCSCI algorithm listed by the percentage of participants responding to that survey question. **A** Value rating of algorithm website functions, *N* listed individually per function evaluated. **B** Impacts of algorithm use, *N* listed individually per impact evaluated. **C** Frequency of participant algorithm use, *N* = 70. **D** Goals of algorithm use, *N* = 64, participants could choose more than one option therefore total does not equal 100%.

User satisfaction, captured in the Praxis User Survey, reflects the majority of participants who used the algorithm (92%, 59/64; *N* = 76, 5 missing, 7 had not used) felt it was very useful to their work with the two most highly valued functions being the automated classification according to the most recent ISNCSCI rules, and the ability to save a .pdf-file of an exam (Fig. 3A). Furthermore, the algorithm increased awareness and use of the ISNCSCI exam, enabled the participants to feel confident in classifying an ISCNSCI assessment, and provided them with support for questions about conducting and classifying their assessments (Fig. 3B).

### Use

The primary algorithm use reported in publications for both EMSCI and Praxis algorithms was to ensure ISNCSCI data accuracy in clinical research, including both clinical trials and observational research using SCI egistries. The EMSCI algorithm has also been incorporated into the EMSCI database (Table 1). The Praxis algorithm has been integrated into the RHSCIR, Australian Spinal Cord Registry, New Zealand Spinal Cord Injury Registry, Dutch National SCI Data Set, and Model Systems Database [31]. Additionally, the EMSCI algorithm was used as a screening tool for the Nogo Inhibition in SCI clinical trial (NISCI) [32], and the ongoing Canadian-American Spinal Cord Perfusion Pressure and Biomarker Study reports using the Praxis algorithm to confirm classification accuracy (Reichl personal communication Nov.2020) [33].

Additional uses reported were integration into electronic medical records (EMRs) and education. The Praxis algorithm has been integrated into EMRs in Denmark (project to incorporate International SCI datasets into EPIC (Verona, Wisconsin, USA) EMR), Finland, Mexico, Korea, and the USA [34]. The EMSCI algorithm was integrated into EMRs of 4 European SCI centers and has been used to evaluate clinical classification skills and the impact of clinical training [6, 14].

How clinicians were using the algorithm was highlighted through the Praxis User Survey (*N* = 76) which included participants from 27 countries. Most were clinicians (69/71, 5 missing) with the majority (78%, 56/72, 4 missing) working in a hospital setting. The survey found that over half of the participants using the algorithm (*N* = 69) did so on a regular basis (51%, Fig. 3C), with 54% (34/63) having incorporated it into their regular workflow. The most common uses were to confirm the classification after the assessment has been completed and to educate others (Fig. 3D). An example specified in the comments outlined this was done by, “using the algorithm to fill the form, check final assessment and include it in patients’ documentation” and “I always have them (students or new staff) calculate the score on their own and then use it as a double check so then we can discuss why the difference”. Other comments mention, “providing a copy to the patient to track progress over time” or using it to, “motivate for continued physiotherapy rehabilitation”. Of those who had used the algorithm, one-third (33%, 22/66) identified having no challenges with using the ISNCSCI algorithm while others reported challenges with internet access (17%, 11/66) and the inability to use it on their smartphones (17%, 11/66).

These algorithms have also been used to inform ASIA’s International Standards Committee where areas identified during algorithm development that would benefit from additional clarification were brought forward for discussion. This has led to the addition of standardized levels for documentation of non-key muscles to the ISNCSCI worksheet and the eighth edition updates on how to classify non-SCI conditions, with other areas still under consideration by the committee. When combined with large clinical datasets, the use of ISNCSCI algorithms allows the ASIA International Standards Committee to evaluate the impact of changes to the ISNCSCI classification rules and make evidence informed decisions. One example of this is the use of the EMSCI ISNCSCI calculator in combination with the EMSCI dataset to inform the updated 2019 ZPP rule change [35].

## Discussion

The use of computerized ISNCSCI algorithms has been around for many years but many were developed and used internally for specific projects or not maintained [7, 10–13]. Today the International SCI community has free access to the updated online format of the EMSCI and Praxis algorithms.

A key reason why these two algorithms are broadly used by the SCI community is their support and integration into large research networks, which in contrast to other algorithms developed, ensures the long-term provision of updated and accurate tools by their developers. Other reasons include the very important initial validation which is the prerequisite for high classification accuracy. For this step, a substantially large dataset is mandatory so that many types of cases can be considered by the algorithm as well as by SCI/ISNCSCI experts, who clarify how to interpret the ISNCSCI rules correctly. Registries like EMSCI and Praxis have broad inclusion criteria which ensures that typical ISNCSCI cases, including cases with classification challenges such as not testable scores, are used for development and validation with human experts. After achieving this milestone, the public interfaces must be developed and maintained, which requires further long-term resources. Finally, the adoption of new ISNCSCI revisions requires substantial resource, which ongoing registries are more likely to provide than specific research projects.

The EMSCI and Praxis algorithms have different interfaces, features, and levels of integration ability for other projects including databases and EMRs and have been and will continue to be developed independently.

One of the big advantages of independent developments is the identification of cases in which they arrive at different classification results (Fig. 2A, C). Both teams collaborate fruitfully on the scientific level, e.g. to identify a problem in the motor level definition to be addressed in future ISNCSCSI revisions [23]. This helps to inform ASIA’s International Standards Committee about the potential need for clarification or correction of certain aspects of ISNCSCI.

Osunronbi recommends that, “Utilizing ISNCSCI calculators can reduce classification errors and may help clinicians with simple but time-consuming tasks … clinicians should not rely exclusively on the ISNCSCI calculators, as human experts may be better than computational algorithms at dealing with complex cases of ISNCSCI classifications such as the presence of non-SCI conditions, and multi-level SCI“, and indeed this is a limitation of computerized ISNCSCI classification algorithms [27]. Although these algorithms can reduce classification errors, they can only be as accurate as the bedside exam scores entered, and cannot provide accurate classification in cases where complex clinical reasoning is required. ASIA’s International Standards Committee has recently emphasized the necessity of well-trained clinical assessors to ensure correct classifications [36]. Both web applications clearly outline this limitation, recommending classification still be performed or reviewed by a skilled examiner, though they continue to improve in the types of cases they are able to classify, with updates to reflect changes introduced with the eighth edition of ISNCSCI, and facilitate reclassification of exams using the updated standards. These algorithms share many similarities and have been successful in being broadly used internationally in both clinical care and research.

The first metric of reach identified a broad number and type of algorithm users with many accessing them using the indirect web application interfaces. This reflects the challenges many have in performing the classification component of the ISNCSCI exam. Clinicians require a simple, easy to access tool to support this skill and researchers require a tool to enable flagging of exams that may have been erroneously classified. This is further supported by Armstrong’s evaluation of ISNCSCI worksheet classification by trained clinicians in three multicenter randomized control trials, who concluded that, “continued training and a computerized algorithm are essential to ensure accurate scoring, scaling and classification of the ISNCSCI and confidence in clinical trials” [26].

For the second metric, usefulness, a key feature is looking at the validation of the algorithms themselves in improving accuracy of clinical classification. Multiple studies that have used these validated ISNCSCI algorithms to evaluate assessor accuracy have shown significant error rates in manual classification [14, 26, 27]. Armstrong reported one or more errors on 74.5% of worksheets across three clinical trials, with errors mostly involving incorrect motor (30.1%), sensory levels (12.4%), ZPP (24.0%) and AIS (8.3%) [26]. Schuld et al. reported on the results of a retrospective computerized reclassification of 420 manually classified ISNCSCI exams and found the lowest agreement in motor levels (62%), motor ZPPs (80.8%) and AIS (83.4%) with AIS B most often misinterpreted as AIS C and vice versa (AIS B as C: 29.4% and AIS C as B: 38.6%) [14]. In a neurosurgical unit where senior clinicians provide formalized but not standardized ISNCSCI orientation training to junior doctors, Osunronbi found an error rate of 17.7% (*N* = 249) in senior clinicians which may have led to higher error rate in the more junior clinicians they provided training to (30.2%, *N* = 119) [27]. Though this is not the ideal ISNCSCI training structure, it accurately reflects the real-world scenario at many hospitals. These studies suggest that nonexperts should receive proper training before using the ISNCSCI in clinical practice, but also highlight the usefulness of validated computer based ISNCSCI algorithms as an additional tool to improve classification accuracy even for trained clinicians.

Perceived usefulness, reported by algorithm users reflects that the ISNCSCI algorithm was also useful in significantly increasing their awareness and use of the ISNCSCI, improving their understanding of the classification rules, ability to assess and classify exams, and also their perceived confidence in classifying. Being confident is one of the most important personal factors influencing clinical decision making and successful assessment [37].

The final metric, use, reflects implementation of the algorithms, and three themes emerged. The first theme, use for education, is shown by the Praxis User Survey which demonstrated that the Praxis ISNCSCI algorithm is used to learn the ISNCSCI classification rules and for educating others. Due to the heterogeneity and complexity of SCI, the ISNCSCI exam is complex, with both theoretical and hands on training required to become competent. ASIA provides many tools to support training (International Standards Training e-Learning Program (INSTeP), ISNCSCI booklet, motor/sensory exam guides), but none of these tools provides real-time exam-specific feedback on classification and support for questions. In a review of trainee perception of medical training technologies, web-based learning was perceived as most valuable when associated with real-time feedback, a simple interface, and extended time for completion, with E-learning interventions that are perceived as lacking interactivity being viewed less favorably [38]. This aligns with the features rated as valuable by respondents (ask questions about a classification they do not understand and access to support for conducting and classifying an ISNCSCI assessment) and represents an area for potential enhancement by making the computational decision process more transparent. Algorithm-supported education in combination with hands-on training and existing tools provided by ASIA, comprise a comprehensive training package.

The second theme, the need for algorithms to ensure data quality, is evidenced by the extensive use of these algorithms both through the publicly available web applications as well as through integration into other registries, databases, clinical trials, and EMRs. Maintaining a high level of quality of ISNCSCI examinations is essential in clinical trials where the classification is often used as inclusion/exclusion criteria, to stratify groups, and as a primary outcome. It is also of utmost importance within networks like EMSCI and Praxis. The use of a standardized computer program to accurately classify ISNCSCI datasets allows clinical trials an additional data quality check, where discrepancies between clinical classification and computer calculated classification can be verified with study sites. It also allows networks like EMSCI and Praxis to ensure high data quality and provide education on classification to their network sites. The differences seen in types of use reported by the scientific literature versus the Praxis User Survey may relate to the fact that the former is probably biased to reflect a researcher perspective while participants of the latter were mainly clinicians.

Interestingly, the third theme was the variety of unintended uses found. These included informing the ASIA International Standards Committee, supporting clinical documentation, conducting bedside exams, and using the resulting worksheet to improve patient self-tracking and motivation. Given the wide variety of unintended uses, future research may be warranted to further explore and engage patients and clinicians to determine their needs and the value of additional features as well as actual demand by these users.

There are several limitations associated with this work which must be considered. Metrics for the evaluation were based on citations using Google Scholar which relies on the authors to include the citation. There may be other studies that used these algorithms without referencing them, resulting in under-reporting use. There is no standardized comprehensive evaluation of both algorithms available so some results are generalized. The Praxis algorithm user survey was conducted on a sample of convenience and was posted on the algorithm web application, which could bias the results. A prospective formal evaluation of both algorithms, targeting centers known to treat individuals with SCI to determine ISNCSCI algorithm use, would be helpful to better understand the breadth of use and inform future enhancements. Future activities planned for the EMSCI and Praxis algorithms include continuing to enhance features for users (e.g. development of an iOS/Android app to address identified limitations of internet access and smartphone compatibility) as informed by how these algorithms are being used and user feedback. A key future direction to be considered by both algorithms will be investigating appropriate ways to incorporate the new Expedited–ISNCSCI which is an abbreviated ISNCSCI designed for use by trained clinicians in screening and follow-up scenarios [39].

In conclusion, the use of validated, computerized classification tools is an effective way to decrease ISNCSCI classification errors due to human error and ensures a consistent set of classification rules is clearly defined. Computerized ISNCSCI algorithms will never replace the role of well-trained clinicians in ISNCSCI classification. They allow reclassification of ISNCSCI datasets with updated versions of the ISCNSCI, and support rapid classification of large datasets. They will continue to support the ASIA International Standards Committee in evaluating the impacts of possible future revisions to make evidence-informed modifications and highlight classification rules which may need further clarification. These algorithms have evolved to be used around the world as a valuable tool to support education, clinical documentation, communication between clinicians and their patients, and ISNCSCI data quality.

## Supplementary information

Supplementary Material

## Data Availability

The datasets generated and/or analyzed during the current study are available from the Praxis Spinal Cord Institute on reasonable request.
