# Supplementary material for: Computer International Standards for Neurological Classification of Spinal Cord Injury (ISNCSCI) algorithms: a review
Source: Spinal Cord. 2022 Sep 16;61(2):125–32. doi: 10.1038/s41393-022-00854-2 (PMC9970871; doi:10.1038/s41393-022-00854-2)
Supplement: Supplementary file 1 — Supplementary Material [file 41393_2022_854_MOESM1_ESM.docx]

**Computer International Standards for Neurological Classification of Spinal Cord Injury (ISNCSCI) Algorithms: A review**

**Supplementary Information**

Kristen Walden (1), Christian Schuld (2), Vanessa K. Noonan (1, 3), and Rüdiger Rupp (2)

1. Praxis Spinal Cord Institute, Vancouver, BC Canada
2. Heidelberg University Hospital, Spinal Cord Injury Center, Heidelberg, Germany
3. International Collaboration on Repair Discoveries, Vancouver, BC Canada

Corresponding author: Rüdiger Rupp (ruediger.rupp@med.uni-heidelberg.de)

**RHI ISNCSCI Algorithm Survey**

1. What is your profession? (check all that apply)

| - Surgeon - Type:______________ |
| --- |
| - Physiatrist (PM&R) |
| - Resident - Type:______________ |
| - Fellow - Type:______________ |
| - Physical Therapist - Occupational Therapist - Registered Nurse - Researcher |
| - Research Coordinator |
| - Other (please specify): ___________________________ |

1. What type of setting do you work in? (check all that apply)

| - Hospital - Private Clinic - Research Institute - Other (please specify): ____________________________ |
| --- |
|  |

1. How do you access the ISNCSCI Algorithm?

- Through the website ([www.isncscialgorithm.com](http://www.isncscialgorithm.com))
- Through a database it has been integrated into
- Through an EMR it has been integrated into
- Other (please specify): _____________________________

1. Approximately how often do you use the ISNCSCI Algorithm?

| Never used it *(respondents then only asked to answer questions 11 , 13 and 15)*  Used it a few times  Use it regularly  Daily  Weekly  Monthly |
| --- |

1. Which of the following have you used the algorithm for? choose all that apply

| - ­­­­­­­­­­­­­­­­­­­­­­­To learn about the ISNCSCI classification rules - To educate others on the ISNCSCI exam - To conduct bedside ISNCSCI exams with patients - To confirm classification of ISNCSCI exams already completed - To check data quality or flag possible incorrect ISNCSCI classifications - For research - To save or print ISNCSCI exam worksheets |
| --- |
| - Other (please describe): _____________________ |

1. Overall, how useful is the Algorithm for your work?

- Very useful
- Somewhat useful
- Not useful

1. What impact if any, has the ISNCSCI Algorithm had on the following:

|  | Significantly Increased | Moderately increased | No impact |
| --- | --- | --- | --- |
| Your awareness of the ISNCSCI |  |  |  |
| Your ability to conduct and classify the ISNCSCI assessment |  |  |  |
| Your confidence in classifying an ISNCSCI assessment |  |  |  |
| Your use of the ISNCSCI assessment |  |  |  |
| Your understanding of the ISNCSCI classification rules |  |  |  |
| Your access to support for conducting and classifying an ISNCSCI assessment |  |  |  |

1. Has the ISNCSCI Algorithm been incorporated into your regular workflow?

Yes No (if no, skip to question #9)

Please describe how it has been incorporated: ______________________________________

1. When you use the ISNCSCI Algorithm, how many ISNCSCI exam calculations do you normally perform?

| - 0-5 | - 16-20 |
| --- | --- |
| - 6-10 | - 21 or more |
| - 11-15 |  |

1. Please rate the value of each of the following ISNCSCI Algorithm functions:

|  | High Value | Some Value | No Value | N/A, I don’t have access to this function |
| --- | --- | --- | --- | --- |
| Automated classification according to the most recent ISNCSCI rules |  |  |  |  |
| Ability to print an exam on the most recent ISNCSCI worksheet |  |  |  |  |
| Ability to save a pdf of an exam |  |  |  |  |
| Ability to classify despite having weakness or sensory change above the level of injury due to something other than SCI (e.g. peripheral nerve injury) |  |  |  |  |
| Ability to see visual representation of sensory scores (dermatome man) |  |  |  |  |
| Ability to ask questions about a classification I don’t understand |  |  |  |  |
| Other functions you find valuable (specify):_____________________ |  |  |  |  |
| Other functions you find valuable (specify): ____________________ |  |  |  |  |

1. If you have not used the ISNCSCI Algorithm, or use it less than you want to, please let us know if there is any particular reason why you have not used it or use it less than you would like to.
2. Please indicate if you have experienced any of the following challenges in using the ISNCSCI Algorithm: (check all that apply)

| - Inability to access the internet - Lack of access to computers - Unable to use on my smartphone - Unclear instructions on how to use the Algorithm - Unable to find information on the Algorithm website - Look and feel of the website is not user friendly - Unable to access completed form in an alternative (e.g. word or excel) format. Format I would like is:___________________________ - Unsure about how to incorporate it into my EMR or research database - Not available in my primary language. My primary language is:___________________ |
| --- |
| - Other (please describe): __________________________________________ - No challenges - Have not used it |

1. Are there other similar tools that you use? *(Note: Information from this question was not included in this publication)*

Yes No (if no, skip to question #13)

If yes,

1. Please describe the tool and provide website address if available.
2. Why do you use other tools in addition to the Algorithm?
3. How could the algorithm be improved?
